# Supplementary material for: Smooth Interpolating Curves with Local Control and Monotone Alternating Curvature
Source: Comput Graph Forum. 2022 Oct 6;41(5):25–38. doi: 10.1111/cgf.14600 (PMC9827861; doi:10.1111/cgf.14600)
Supplement: Supplementary file 1 — Supplement Material [file CGF-41-25-s001.zip › Local-Smooth-Interpolating-MonoCurvature/extern/clothoids/docs/references.html]

References — Clothoids v2.0.9

### Navigation

- index
- toc
- Clothoids »
- References

# References¶

1. *E. Bertolazzi, M. Frego*, **G1 fitting with clothoids**,

   Mathematical Methods in the Applied Sciences,

   John Wiley & Sons, 2014, vol. 38, n.5, pp. 881-897,

   https://doi.org/10.1002/mma.3114
2. *E. Bertolazzi, M. Frego*, **On the G2 Hermite interpolation
   problem with clothoids**,

   Journal of Computational and Applied Mathematics,

   2018, vol. 15, n.341, pp. 99-116.

   https://doi.org/10.1016/j.cam.2018.03.029
3. *E. Bertolazzi, M. Frego*, **Interpolating clothoid splines with
   curvature continuity**,

   Mathematical Methods in the Applied Sciences,

   2018, vol. 41, n.4, pp. 1099-1476.

   https://doi.org/10.1002/mma.4700
4. *E. Bertolazzi, M. Frego* **Point-Clothoid Distance and Projection Computation**,

   SIAM Journal on Scientific Computing,

   2019, 41(5), A3326–A3353.

   https://doi.org/10.1137/18M1200439
5. *E. Bertolazzi, M. Frego* **A Note on Robust Biarc Computation**,

   Computer-Aided Design & Applications 16 (5), 822-835

   http://www.cad-journal.net/files/vol\_16/CAD\_16(5)\_2019\_822-835.pdf

### Quick search

### Table of Contents

- Matlab Interface Manual
- C++ API
- MATLAB API

«
hide menu

menu
sidebar
»

### Navigation

- index
- toc
- Clothoids »
- References

© Copyright 2021, Enrico Bertolazzi and Marco Frego.
Created using Sphinx 4.2.0.
